# Tumour Stem Cells in Breast Cancer

**DOI:** 10.3390/ijms23095058

**Published:** 2022-05-02

**Authors:** Marina Ibragimova, Matvey Tsyganov, Nikolai Litviakov

**Affiliations:** 1 Cancer Research Institute, Tomsk National Research Medical Center, Russian Academy of Sciences, 5, Kooperativny Street, 634050 Tomsk, Russia; tsyganovmm@yandex.ru (M.T.); nvlitv72@yandex.ru (N.L.); 2 Laboratory of Genetic Technologies, Siberian State Medical University, 2, Moscow Tract, 634050 Tomsk, Russia; 3 Biological Institute, National Research Tomsk State University, 36, Lenin, 634050 Tomsk, Russia

**Keywords:** cancer stem cells, breast cancer, metastasis, plasticity

## Abstract

Tumour stem cells (CSCs) are a self-renewing population that plays important roles in tumour initiation, recurrence, and metastasis. Although the medical literature is extensive, problems with CSC identification and cancer therapy remain. This review provides the main mechanisms of CSC action in breast cancer (BC): CSC markers and signalling pathways, heterogeneity, plasticity, and ecological behaviour. The dynamic heterogeneity of CSCs and the dynamic transitions of CSC^−^ non-CSCs and their significance for metastasis are considered.

## 1. Introduction

All types of malignant neoplasms (MNOs) consist of different populations of tumour cells that give the tumour the property of heterogeneity. Tumour stem cells (CSCs) play a key role in tumour initiation, maintenance of tumour growth and progression [1]. CSCs demonstrate all aspects of stemness. However, despite the physiological role of adult tissue stem cells (SCs), they are unable to maintain tissue homeostasis due to uncontrolled division [1].

Tumour stem cells are a small population of cells in a tumour with stem-like properties that support an increased capacity for self-renewal, growth, metastasis, and reproduction, which causes cancer progression. CSCs can regulate neighbouring cells, supply them with nutrients, create an environment for tumour growth, or vice versa, and use them for their survival by the mechanisms of induction of autophagy and entosis [2]. CSCs form heterogeneous cell populations, most often with a high plasticity potential [2] and high resistance to stress factors within the tumour microenvironment and cell death induction under the influence of chemotherapeutic drugs—resistance to antitumour therapy [3]. Breast tumour stem cells (CSCs) exist in the form of a small fraction of cells in the mammary gland; they are undifferentiated and can produce new CSCs through symmetric division and, through asymmetric division, produce tumour cells that differentiate and leave the main population of tumour cells [4]. It is assumed that asymmetric divisions lead to the appearance of first generation progenitor cells capable of limited proliferative activity [5,6]. It is believed that the functioning of CSCs occurs in close interaction with their specific microenvironment, the breast stem cell niche, which CSCs themselves regulate [7]. The locations of these cells, their unique characteristics of self-renewal, differentiation, and plasticity, and their role in breast cancer are still the subject of discussion. This review is devoted to the analysis of current data on tumour stem cells in breast cancer.

## 2. Cancer Stem Cell Signalling Pathways

Many mutations in tumours are involved in the activation of cell self-renewal pathways, including in CSCs. In cancer, multiple cellular self-renewal pathways are not only continuously activated but can also be amplified. The activation of the self-renewal programme is an important part of the stem nature of CSCs, promoting tumour progression and metastasis, causing high cell turnover and the production of progenitor cells [2].

First, we concentrate mainly on the data currently published in the world literature about signalling pathways that promote self-renewal of stem and/or progenitor cells, the dysregulation of which can contribute to oncogenesis.

### 2.1. Canonical WNT/Β-Catenin Signalling

WNT signalling is activated through an autocrine mechanism in breast cancer. It has been shown that in approximately 50% of clinical cases of breast cancer, there is a high level of stabilised β-catenin, as well as a high frequency of amplification of the positive genes stimulating the WNT-pathway [8]; moreover, when the frequency of amplification of positive stimulating of WNT signalling exceeds the frequency of amplification of negative regulators of WNT, this is combined with the aggressive course of breast cancer [9]. Protein FRP1 (WNT inhibitor) is deleted in 78% of breast cancer cases, and its deletion is associated with a poor prognosis [10]. Activated β-catenin promotes triple-negative and HER2^+^ breast cancer [11]. In this regard, the restoration of negative WNT regulators, which are not expressed in the tumour due to methylation of promoters or deletion of genes encoding negative WNT regulators in the tumour, has become a new direction of therapy. There is evidence that the restoration of negative regulators effectively slows the growth of experimental tumours [12]. Inhibition of WNT1 changes the phenotype of CD44^+^CD24^−^ALDH1 stem cells and reduces their ability to form tumours and cell migration [13], and suppression of GSK3/β-catenin signals by an inhibitor of protein kinase D1 (PRKD1) is sufficient to reduce the stem and chemoresistance of breast cancer cells [14]. Modulation of the nuclear-antigen-associated factor RAF of proliferating cells in mammary CSCs by NVP-AUY922 suppresses their capacity for self-renewal and heterogeneity [15]. Gujral et al. also found that the levels of WNT5A/B and its Frizzled2 receptor are elevated in tumours and in several breast tumour cell lines. Frizzled2 induces epithelial–mesenchymal transition (EMT) in a noncanonical pathway, stimulating stemness [16].

### 2.2. Notch Signalling Pathway

Notch3 and Jag1 are key regulators of CSC renewal and survival during hypoxia in breast cancer and tumours derived from breast cancer cell lines. Notch expression is significantly increased in CSCs during hypoxia and leads to resistance to inhibition of the PI3K/mTOR pathway [17]. The Notch pathway plays a role in increasing the number of CSCs through symmetric division [18] and contributes to the dedifferentiation of progenitor cells by stimulating EMT [19]. At the same time, inhibition of Notch1 by specific antibodies significantly reduces the subpopulation of CSCs with the CD44^+^ CD24 phenotype and the frequency of brain metastases in breast cancer [20]. Violation of the negative regulation of Notch signalling initiates the development of several types of cancer, including breast cancer [21]. Notch-positive cells show a better possibility of tumour initiation than Notch-negative cells [22].

### 2.3. Hedgehog (HH) and Sonic Hedgehog (SHH) Signalling Pathway

Activation of the Hedgehog signalling pathway is associated with the development of several types of cancer, including breast tumours [23]. Hh signalling pathways regulate self-renewal in breast stem cells [24], and destruction of lower transcriptional targets of the SHH, PTCH-1, or GLI-2 pathways leads to severe defects in mammary duct morphogenesis [25]. SHH activation may contribute to relapse and can be used as a predictor of postoperative relapse in breast cancer. High expression levels of SHH, PTCH-1, GLI-1 and SMOH correlate with the invasiveness of breast tumour cells [26]. The transmembrane protein Patched (PTCH) is a receptor of the hedgehog signalling molecule family (Sonic-Shh, Indian-Ihh, and Desert-Dhh) and is associated with early embryonic tumorigenesis. PTCH inhibits the activity of the Hh pathway through its interaction with the Smoothened transmembrane protein (SMO). Overexpression of SHH, PTCH1, and GLI1 is found in most cases of breast cancer [19,27]. Evidence continues to mount and to demonstrate the role of the Hh pathway in the development of metastasis, particularly through the initiation of EMT [28] and the activation of proteins such as MMP-9 and the expression of E-cadherin [29].

### 2.4. NRF2 Signalling

The NRF2-mediated antioxidant pathway is a novel mechanism that explains the chemo- and radioresistance of tumour stem cells. Significantly higher expression of NRF2 and target genes of this pathway was found in CSCs than in normal breast cells. NRF2 suppression delays mammosphere formation and changes therapy-resistant phenotypes in MCF-7 breast cells [30]. NRF2 can push dormant tumour cells to proliferate or bypass metabolic blocks [31]. Tumour cells generate more reactive oxygen species (ROS) than normal cells, and CSCs require low ROS levels to maintain dormancy and self-renewal. Combining chemotherapeutic agents with CSC antioxidant capacity makes tumours vulnerable to chemotherapy and radiotherapy, while normal tissues are practically unaffected. For this purpose, various natural inhibitors of NRF2, such as apigenin, ATRA, all-trans retinoic acid, brusatol, chrysin, cryptotanshinone, luteolin, trigonelline, and и wogonin have been tested in preclinical tumour models [32]. Unfortunately, the exact mechanisms of the inhibitory effect on NRF2 are poorly understood.

### 2.5. PI3K/AKT/mTOR Pathway

The activation of PI3K signalling in breast cancer has been frequently noted in the last few years in the literature and is mainly due to genetic mutations. For example, mutations in the tyrosine kinase receptor can aberrantly increase PI3K activity [33], and loss of PTEN function is found in approximately 50% of breast cancer patients [34]. PIK3CA mutation triggers centrosome amplification and increases tetraploidization-tolerance in breast cancer cells [35]. PIK3CA mutations induce dedifferentiation of progenitor tumour breast cells into tumour stem cells [36].

The PI3K/Akt/mTOR signalling pathway is associated with metastases in CSCs. Stable stemness is partially dependent on PI3K-regulated transactivation of several self-renewal pathways, including Wnt/β-catenin in triple-negative breast cancer [37]. Inhibition of the PI3K signalling pathway to inhibit tumour growth is not a new idea. However, taking into account the dualism of the PI3K/Akt/mTOR pathway, inhibition of PI3K by a buparlisib inhibitor may stimulated the Wnt pathway, which appropriates a stem-like phenotype in triple-negative breast cancer cells [37]. In addition, PI3K inhibitors can stimulate GLP-1-dependent stemness in MDA-MB-231 and MCF-7 breast cancer cell lines [38].

**Figure 1:**
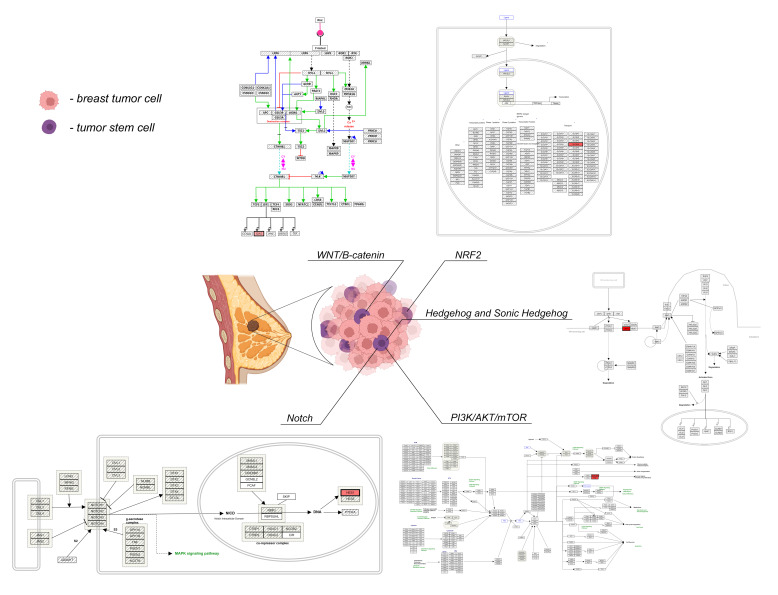
The signalling pathways of CSCs in breast cancer. Note: Created with Biorender.com. Wnt signaling pathway (Homo sapiens). https://www.wikipathways.org/index.php/Pathway:WP363 (accessed on 1 March 2022). Notch signaling (Homo sapiens). https://www.wikipathways.org/index.php/Pathway:WP268 (accessed on 1 March 2022). NRF2 pathway (Homo sapiens). https://www.wikipathways.org/index.php/Pathway:WP2884 (accessed on 1 March 2022). Hedgehog signaling pathway (Homo sapiens). https://www.wikipathways.org/index.php/Pathway:WP4249 (accessed on 1 March 2022). PI3K-Akt signaling pathway (Homo sapiens). https://www.wikipathways.org/index.php/Pathway:WP4172 (accessed on 1 March 2022).

The signalling pathways regulating the functional activity of CSCs in breast cancer are schematically shown in Figure 1.

## 3. Markers and Heterogeneity of CSCs

The following markers are allocated as breast CSC markers: CD44, CD24, CD133, EpCAM, nestin, GD2, CD49f, CD61, CXCR4, CXCL1, HMGCS, CD166, CD47, ALDH1, and ABCG2 [39,40,41,42,43,44,45,46,47,48,49,50,51,52,53,54,55,56,57,58,59,60,61]. CSCs were first identified and isolated in a xenograft model obtained from a patient with breast cancer in 2003 [40]. The oncogenic subpopulation of cells contained the surface marker CD44^+^CD24^−/low^. In the following few years, such subpopulations were found in early disseminated cells from the bone marrow of patients with breast cancer and were associated with the occurrence of relapse and distant metastasis [41]. Undifferentiated CD44^+^CD24^−/low^ cells in tumours after chemotherapy were associated with poor outcomes in patients with invasive breast cancer. In addition, an increase in the proportion of CD44^+^CD24^−/low^ cells in the tumour was associated with lymphogenous metastasis [42]. These data suggested the importance of CSCs in the relapse and metastasis of breast cancer. However, CD44^+^CD24^−/low^ cell markers are not universal CSC markers. For example, in MDA-MB-231 and MDA-MB-361 cell lines, most cells show a CD44^+^CD24^−/low^ phenotype, but only 5% and 12% of them, respectively, have oncogenic ability [43,62]. The correlation between an increase in the proportion of CSCs in breast tumour tissue and a poor prognosis increases when ALDH1 is used in combination with the CD44^+^CD24^−/low^ phenotype [44].

It is important to understand that one of the serious and long-standing problems in the study of CSCs is determining the appropriate methodology for the isolation and characterization of cells [45]. The main method for CSC identification is cell-sorting based on the expression of cell surface markers, such as CD44, CD133, CD24, CD26, EPCAM, and CD166, or based on the enzymatic activity of intracellular proteins, such as ALDH1 [46]. However, these markers are not universal and are not expressed on all CSCs, which limits their use. To overcome this limitation, it is necessary to use several markers [45]. Possible CSC phenotypes identified in breast cancer are shown in Table 1.

CSCs with the CD44^+^ phenotype are closely associated with metastasis. The proportion of early disseminated tumour cells with the CD44^+^CD24^−^ phenotype in the bone marrow of patients with breast cancer was approximately 72% [68]. Labelled nanoparticles to target CD44^+^ CSCs have shown great potential for the capacity to strengthen the effect of chemotherapy in vitro [69]. The occurrence of spontaneous metastasis to the lungs and lymph nodes during transplantation of CD44^+^ CSCs in mice was shown by using noninvasive imaging methods [70]. The integrins CD29, CD49f, and CD61 are effective markers for CSCs. CD49f cells in breast tumours are associated with an increased possibility of metastasis and shorter survival time in patients [71]. Subpopulations of tumour cells that were resistant to doxorubicin and paclitaxel had CD49f^+^ and CD61^+^ phenotypes in Her2^+^ primary mammary tumours in mice [57]. The exact role of the CD133 transmembrane protein in breast cancer is unclear. However, BC cells with a CD133^+^ phenotype have stem-like properties. MDA-MB-231 cells with the CD133^+^ phenotype have a higher colony-forming efficiency than CD44^+^ cells [51]. It was also found that the number of CD133^+^ cells increases in tumours in patients with hormone-resistant breast cancer, contributing to the occurrence of metastasis regardless of the ER status of patients [52]. Exosomes can transfer miR-221 to breast tumour cells and contribute to Notch3 activation, which is required for CD133 cell proliferation [72].

The NAD(P)^+/−^ dependent enzyme ALDH1 acts as an independent predictor of poor survival in breast cancer patients [73]. MDA-MB-231 and MDA-MB-468 cells with the CD44^+^CD24^−^ALDH1^+^ and CD44^+^CD133^−^ALDH1^+^ phenotypes had a stronger oncogenic and metastatic ability than ALDH1^−/low^CD44^−/low^ cells [64]. Analysis of mammosphere formation has shown that ALDH blockade increases the proportion of CSCs in several breast cancer cell lines, including 184A1, SUM149, SUM159, and HCC1954 [74].

Mukherjee et al. investigated CXCR4 (membrane chemokine receptor). The authors found that nonmigrating CSCs contributed to the transformation of non-stem tumour cells to metastatic CXCR4^+^ cells in primary breast cancer tissue. The results not only pointed out the potential of CXCR4 as a marker of breast CSCs but also provided evidence for the transformation of non-stem tumour cells to stem cells. The decrease in E-cadherin and increase in vimentin suggest that they underwent EMT dedifferentiation [53]. CXCR4 hyperactivation is closely related to changes in the tumour microenvironment. Activation of SDF-1/CXCR4 signalling may increase the phosphorylation of 60 proteins associated with the migration and invasion of CD44^+^CD24^−^ CSCs in breast cancer [75].

The ABCG2 protein is highly expressed in several chemoresistant breast cancer cell lines. Compared to non-stem cells, CD44^+^CD24^−/low^ cells from MCF-7, MDA-MB-231, and SK-BR-3 breast cancer cell lines showed higher expression of ABCG2 [59]. Higher expression of the endothelial marker ANTXR1 was found on the cell surface of CD44^+^CD24^−^ and ALDH1^+^ of the TMD231 breast cancer cell line. Overexpression of ANTXR1 activates key genes for cell proliferation, DNA replication, and WNT signalling pathways, giving increased tumorigenic and metastatic potential to breast cancer cells. Moreover, ANTXR1 partially mediates the induction of tumour cell stemness. The authors argue that it is possible to sort the subpopulation of malignant breast cancer CSCs by the presence of ANTXR1 [48]. EpCAM (CD326 or ESA) plays an important role in the migration and metastasis of tumour cells. EpCAM^+^ circulating tumour cells in breast cancer contain a subpopulation of metastatic cells [41]. PROCR is a specific CSC marker for triple-negative breast cancer. The PROCR^+^ cell lines MDA-MB-361 and MDA-MB-231 have a 2-fold and 9-fold increase in the efficiency of colony formation, respectively, compared to PROCR–cells [43,62]. It was shown that the expression of GD3S is visibly increased in GD2^+^ CSCs of breast cancer. At the same time, GD3S knockdown decreases GD2 expression and disrupts their ability to migrate and form mammospheres, as well as disrupting the initiation and maintenance of EMT [67,76]. Overexpression of GD3S promotes stem properties and metastatic potential in MDA-MB-231, MDA-MB-468, and MCF-7 cell lines [67,76].

Despite the available data on the role of individual markers in determining CSCs in breast cancer, transcriptome analysis showed that many CSC markers can be coexpressed by one cell simultaneously [77], and the expression of CSC markers can change in vivo as a result of plasticity and adaptation to the microenvironment [78]. These observations emphasise the heterogeneity of CSCs and the ineffectiveness of markers used to identify CSCs and non-CSCs [79].

Thus, the CSCs population in BC is heterogeneous and is characterised by the presence of a wide variety of markers associated with stemness properties.

## 4. Ecological Behaviour of CSCs, Autophagy and Enthosis

The consequence of intratumoural heterogeneity is the emergence of various kinds of ecological relationships between CSCs, other tumour cells, and the microenvironment [80]. At the same time, such interactions can be both positive and negative, depending on the internal and external conditions. Tumour cells and the microenvironment observe positive types of interactions (commensalism, mutualism) under favourable conditions between CSCs. For survival, CSCs use negative strategies of ecological behaviour when interacting with their descendants and cells of the microenvironment, such as amensalism in the form of autophagy, parasitism in the form of induction of autophagy of neighbouring cells, predation in the form of cannibalism, and the formation of hybrid heterotypic cells with immunocytes under unfavourable conditions [80].

**Figure 2:**
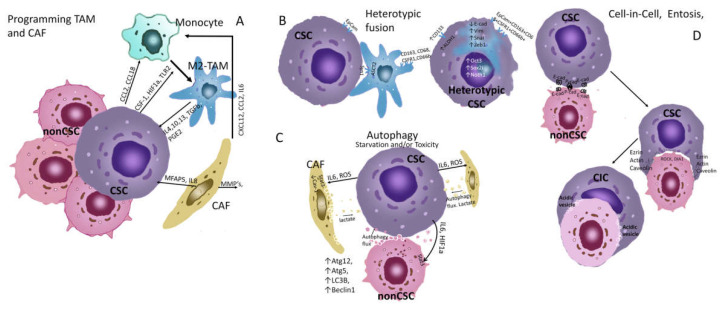
Ecological behaviour of CSCs, autophagy and entosis. (**A**) Programming TAM and CAF. Activation of ERK/STAT3 signaling molecules triggers M2 polarization of monocytes in breast tumours [86]. The recruitment of monocytes from the blood is carried out due to the production of chemokines—CCL2, CCL18, and cytokines CSF1, VEGF, etc., by tumor cells. When polarizing monocytes in M2-TAM CSC, CSF1, HIF1a, TLR2, and other factors are used. [87]. CAF could promote monocyte recruitment toward cancer cells through CXCL12/CXCR4 pathway [88]. CAF produces MMPs that degrade the matrix and promote invasion of CSCs. CAFs produce IL17a which stimulates symmetric division of CSCs [89]. M2-TAMs produce IL4, 10, 13, TGFb, and PGE2, which act on CSCs and stimulate their symmetric division, increasing their population [90]. Thus, this type of relationship can be seen as a commensalism between TAM, CAF, and CSCs. The interaction between TAM and CSCs is described in detail in the overview [91]. (**B**) Heterotypic fusion. Heterotypic fusion cells expressed the tumour marker EpCam and macrophage markers CD163, CD68, CSFR1 and CD66b [92]. The hybrid cells display EMT with a significant downregulation of E-cad and upregulation of N-cad, Vim and Snail, as well as an obviously increased expression of MMP-2, MMP-9, uPA, and S100A4. The TCF/LEF transcription factor activity of the Wnt/β-catenin pathway and the expression of its downstream target genes including cyclin D1 and c-Myc increased in the hybrid cells [93]. This type of relationship can be viewed as a symbiosis with the acquisition of new properties that are not characteristic of individual populations. (**C**) Autophagy. With the induction of autophagy in CSCs, the expression of ATG5-12 LC3B, and BECLIN1, as well as the transcription factors FOXO3A, SOX2, NANOG, and STAT3, which can lead to the induction of the stem phenotype, increased resistance to chemotherapy, EMT, increased migration activity, and survival of dormant cells [94,95,96,97]. Under starvation and toxicity, CSCs can also induce autophagy of neighboring microenvironmental cells and non-CSCs, through exposure to ROS, IL6, and HIF1a. In this case, cells produce metabolites that feed CSCs, in particular lactate [94]. In this case, the behavior of CSCs can be seen as parasitism. (**D**) Cel-in-Cell. E-cadherin and P-cadherin are key components of the adherent junctions of cells in entosis process [98]. These molecules bind CSCs and non-CSCs scavenged. Increased RHO–RHO-associated coiled-coil-containing protein kinase (ROCK) and/or diaphanous-related formin 1 (DIA 1)–actomyosin show in losers cells [99]. CSCs express ezrin, actin, and caveolins, which mediate uptake [100]. Absorbed cells undergo apoptosis, autophagy, or are destroyed by lysosomal digestion, providing CSCs with nutrients [99]. In addition, entosis can lead to the formation of aneuploidy in CSCs [101], and this may be a rapid mechanism for the formation of new chemoresistant clones during treatment.

Positive interactions are widely known (Figure 2a). Tumour cells can carry out M2 polarization of macrophages, which secrete IL-10, TGF-β, and VEGF into the environment, stimulating the growth of breast tumours [81,82]. The MBA-MB-231 cell line with a large number of CSCs produces IL-8 and stimulates the formation of the same interleukin by fibroblasts/macrophages in the case of their joint incubation, which enhances the proliferation and migration of tumour cells [83]. Distant signal transmission is possible due to the paracrine secretion of various biological substances (cytokines, growth factors, microRNA, extravesicles) into the environment [84,85].

Tsuji et al. [102] observed mutually beneficial cooperation of tumour clones initially possessing different biological properties (high metastatic potential but low invasive potential, and vice versa). During coinoculation of tumour clones, animals formed metastases-containing cells with a low metastatic potential, which, in the control, did not form metastases by themselves. CSCs can produce offspring that transdifferentiate and acquire characteristics similar to endothelial cells, forming vascular-like tubular structures [103].

Heterotypic cell fusion is a fundamental developmental mechanism that is well known for normal cells (sperm and egg, fusion of haematopoietic and epithelial cells in response to injury) and is an example of positive cellular interactions or symbiosis [104]. The heterotypic fusion of tumour cells with normal cells can be considered an example of symbiosis. On the one hand, this is a mechanism for the escape of tumour cells from the immune system, including during immunotherapy; on the other hand, the fusion of tumour cells with normal mesenchymal cells is a tool for acquiring a locomotor phenotype. The stem properties of tumour cells are enhanced by heterotypic fusion, and completely new properties are acquired [105]. The circulating hybrid cell (CHC) population (defined as cells with macrophage and epithelial/tumour properties) has been shown to correlate with disease stage and predict disease outcome. These cells expressed the tumour marker EpCam and macrophage markers CD163, CD68, CSFR1, and CD66b (Figure 2). Experimental hybrids of tumour cells and macrophages easily formed liver metastases in mice when injected into the spleen and lungs, i.e., had metastasis-initiating properties. In addition, hybrid clones retained macrophage genotypes with functional phenotypes, thereby imparting macrophage-like behaviour to neoplastic cells [92]. Heterotypic cell fusion most frequently occurs under hypoxic conditions [106]. Recently, there has been wide public activity devoted to the topic of tumour cell hybrids, which reflects that this is an infrequent event. In addition, hybrid cells still undergo post-hybrid selection [93,107,108].

Negative interactions are of great interest; they are much more likely to occur due to unfavourable conditions than positive interactions, and they determine tumour progression after treatment. Autophagy is one of the main mechanisms of negative interactions and has been considered in the past few years as a prerequisite for maintaining stem cells as normal stem cells [109], and CSCs [96,110,111]. The role of autophagy in cancer is multifaceted: it promotes the survival of tumour cells by supplying processed metabolites for growth, modulates mitochondrial function through mitophagy (selective mitochondrial degradation), and participates in the migration and invasion of tumour cells by controlling migrating cytokine secretion [112]. Autophagy plays a central role in the tumour microenvironment [112,113].

Autophagy in CSCs (Figure 2C) increases the expression of stem cell markers such as CD44, as well as the expression of mesenchymal markers such as vimentin. Autophagy also promotes spheroid formation in vivo, which proves its critical role in maintaining CSCs [94,96,112]. Inhibition of autophagy reduces the survival and metastasis of dormant tumour cells in mice [95]. It has been shown that autophagy is required to increase CSC self-renewal in the hormone-independent breast cancer cell line LM38-LP [114]. The autophagy markers Atg5, Atg12 and LC3B are overexpressed in dormant stem cells, such as breast cancer cells, and 3-MA suppression of autophagy removes cells from the dormant state [115]. In 2017, Zhang et al. found that inhibition of autophagy may be partially responsible for the suppression of stem-cell markers in breast cancer [116].

Key transcription factors associated with autophagy induction and stem cell health, such as FOXO3A (which induces the expression of autophagy genes in stem cells), switch themselves by autophagy. Other transcription factors, including the main stemness factors SOX2, NANOG and STAT3, are also associated with the induction of autophagy; they modulate autophagy genes and determine the stemness of CSCs [96,97]. Autophagy allows CSCs to survive despite hypoxia and low nutrient levels in the tumour microenvironment during growth and treatment [94]. Autophagy regulates luminal progenitor-like cells through the TGF-β-Smad and EGFR-Stat3 signalling pathways in MMTV-PyMT breast tumours [117].

Autophagy, which is induced in cancer-associated fibroblasts (CAFs), leads to an increase in the production of amino acids, which are passed through paracrine transmission to support tumour cell growth [118]. The conversion levels of Beclin1 and LC3-II/I proteins in TFA are higher than those in normal fibroblasts in breast cancer, and autophagy of TFA can enhance the proliferation of TNBC cells [119]. In nutrient deficiencies, staining with the autophagic marker Beclin-1 reveals autophagic regions surrounding active proliferating cells [120].

The role of the autophagy effect on CSCs in the antitumour therapy of breast cancer is also discussed. It has been shown that anticancer therapy can induce autophagy, which contributes to the survival of CSCs [121]. Chloroquine, CQ, is an inhibitor of autophagy and causes damage to mitochondria, which leads to excessive oxidative damage to DNA and subsequent death of CSCs TNBC [122]. Inhibition of autophagy can disturb the maintenance of CSCs. Wen Yue has shown that salinomycin is at least 100 times more effective than paclitaxel in reducing the proportion of mammary CSCs, as it can suppress their survival through autophagy [123]. The effects of autophagy blockade on breast cancer CSC activity include suppression of the expression of stem cell factors OCT4, SOX2, NANOG, and CD44; a decrease in the number of mammospheres; an increase in susceptibility to chemotherapeutic agents; and a decrease in the survival of tumour cells and metastasis [114,116,123]. Thus, autophagy can be induced in a tumour and mediate resistance to therapy due to the survival of CSCs in various types of cancer in response to the applied treatment [124,125]. Clinical trials of hydroxychloroquine for targeting autophagy and cancer treatment have been launched [126].

Another interesting type of negative cellular interaction is predation, which manifests itself as a cell-in-cell (CIC) phenomenon (Figure 2D). The first reports describing the presence of cells in other cells in tumour tissues appeared approximately 120 years ago [99,127]. Tumour tissues contained cells with a sickle nucleus because the vacuole displaces the cell nucleus to the periphery. This led to the emergence of the term “cell in a cell” [128]. Cell-in-cell structures can be formed as a result of the activation of cannibalistic mechanisms. They are similar to phagocytosis of macrophages or different actions that involve cell invasion into each other rather than absorption [129].

CICs can be formed by different processes and can be composed of two or more cells of the same origin (e.g., two tumour cells, homotypic) or cells from different origins (e.g., immune cells within tumour cells, heterotypic). Several different terms have been described in the literature to denote the formation of CIC: entosis, uptake, EM teripolysis, and cannibalism [130,131]. The cannibalistic activity of tumour cells is an important metabolic adaptation to an unfavourable microenvironment with a lack of nutrition. Entosis is also characteristic of metastatic cells [99]. Recent studies have highlighted the importance of the CIC phenomenon in the behaviour of many types of cancer [132,133]. The “cell-in-cell” phenomenon for breast cancer is an independent prognostic factor; it is the entotic formations that represent 91% of the CIC structures in the samples of breast ductal carcinoma collected from untreated patients [132]. The entosis process has also been demonstrated in the MDA-MB-231 breast cancer cell line, which cannibalises mesenchymal stem cells at a high rate when mixed in 3D cocultures designed to mimic bone metastases [134]. The anticancer drug paclitaxel induces entosis in the MCF7 breast cancer cell line [135].

Thus, the ecological behaviour of CSCs includes both forms of positive interactions, such as commensalism, mutualism and symbiosis, that appear under favourable conditions for tumour development, and negative forms of ecological behaviour that prevail under unfavourable conditions: amensalism in the form of autophagy, parasitism in the form of induction of autophagy of neighbouring cells, and predation in the form of cannibalism or entosis.

## 5. Phenotypical Plasticity Determines Dynamic Heterogeneity of CSCs

CSCs are dynamic populations and can undergo spontaneous transitions between states and phenotypes [136]. Chaffer et al. showed that cancer non-stem cells spontaneously switch to cancer stem cells in vitro and in vivo by using the example of basal cells of breast cancer. Later, it was discovered that this plasticity is regulated by ZEB1, a key regulator of EMT [137,138].

CSCs form heterogeneous cell populations with high plasticity potential for various forms of cancer [2]. Different markers can identify different CSCs within the same tumour type; they are phenotypically different and can vary from patient to patient depending on the genetic structure of the tumour [139].

CSC BC can be distinguished by epithelial and mesenchymal phenotypes, even if the identification markers are similar. Various subpopulations of CSCs were identified based on the markers ALDH1, CD44, and CD24 and two subpopulations: epithelial-like with the ALDH1^+^ phenotype and mesenchymal-like with the CD44^+^CD24^−^ phenotype. This subpopulation is capable not only of mutual transformation among themselves but also of the formation of non-CSCs [140]. CSCs and non-CSCs show a dynamic balance maintained through cytokine-mediated cross-interactions between populations in breast cancer. Thus, tumour-treatment strategies aimed at destroying CSCs appear to be ineffective [141]. In this regard, therapeutic strategies aimed at blocking phenotypic plasticity, in particular, dedifferentiation of non-stem tumour cells into CSCs, may be promising.

The results show that phenotypic plasticity is reversible and does not necessarily depend on genetic changes [142]. CSC plasticity was also manifested in the formation of tumour pseudoorgans in vasculogenic mimicry, a characteristic process of tumour cell plasticity in which tumour cells transdifferentiate and acquire endothelial cell characteristics [103]. It was shown that a population of cells with the CD133^+^ phenotype with CSC-like properties demonstrated the ability to form vascular-like tubular structures in triple-negative breast cancer [143]. Thus, CSCs can not only mutually transform their subpopulations but also give rise to various types of undifferentiated CSCs and differentiate into new tumour pseudoorganoids.

## 6. CSCs of Primary Tumour and Metastasis

BC predominantly metastasises to the bone (47–60%), liver (19–20%), lung (16–34%) and brain (10–16%) [144,145]. The primary tumour that subsequently metastasises is highly heterogeneous, which makes it difficult to assess risk factors for metastasis [146]. Metastasis or relapse spreads from primary tumours with further acquisition of mutations and independent evolution of metastases, and there are acquired mutations in distant metastases that were not detected in primary tumours [147]. Primary tumours and metastases of various types of cancer are closely related from a genetic point of view, but no genetic changes have yet been found that underpin metastasis [148]. Metastatic cells have the ability to differentiate into CSCs and initiate tumours. On the one hand, metastatic cells can originate from a CSC subpopulation that already undergoes genetic changes to initiate tumour growth [145]. CSCs also have the ability to metastasise due to their tumour-initiating ability [149]; in this case, markers of metastasis should be looked for directly in the CSC subpopulation. On the other hand, metastatic potential can be acquired due to the ability to dedifferentiate to CSCs from non-CSCs directly in the focus of possible metastasis. [150] and in this case, it is not necessary to look for markers of metastasis but, rather, markers of the ability to dedifferentiate.

It is assumed that the latent phase of breast metastasis may include the spread of cells at an early presymptomatic stage of primary tumour development. Disseminated tumour cells remain undetected for many years before they develop into macrometastases and become clinically significant [151]. Disseminated tumour cells with stem-cell properties and in the dormant state can be activated in response to injury during treatment (chemotherapy, targeted therapy, radiation therapy, surgery, etc.), to promote the formation of a new tumour or metastasis [6]. At the same time, cells initiating metastasis can exist in a subpopulation of breast cancer CTCs in mouse models [41].

CTCs obtained from patients with metastatic breast cancer often show overexpression of stem cell markers, suggesting that metastasis is induced by a subpopulation of CTCs that express a tumour stem-cell marker (CD133 or CD44), have CSCs characteristics and thus can be considered as circulating CSCs (cCSCs) [152,153]. Accordingly, CTCs are important objects for understanding carcinoma metastasis [154]. However, it is important to understand the high degree of heterogeneity of circulating tumour stem cells in invasive breast cancer. The work of Savelieva et al. demonstrated significant heterogeneity among CTCs with stem-like features in the form of co-expression variants of CD44/CD24, CD133 and ALDH1 markers. In patients who had CD44^−^CD24^−^ CTCs, a subset of cells with the expression of other stem-cell markers (CD133 and ALDH1) were detected. Expression of CD133 and/or ALDH1 may be associated with expression of N-cadherin; all populations of N-cadherin^+^ CTCs demonstrate stem features [155]. The question remains as to why CSCs under favourable conditions in the primary tumour undergo EMT, begin to migrate, penetrate into secondary organs and form metastases. Unfavourable hypoxia, nutritional deficiencies, etc., do not explain why small tumours can give metastases and do not explain the earlier origin of metastases.

More than 10 years ago, evidence began to emerge that CSCs are cells that mediate tumour metastasis, treatment resistance, and disease recurrence in breast cancer. Breast-tumour gene profiling has shown that CSCs have an invasive gene signature that correlates with increased metastasis and poor overall survival [156]. Baccelli et al. identified the population of tumour cells circulating in the blood from breast cancer patients, which initiates metastasis. The number of CSCs with the EpCAM^+^CD44^+^MET^+^CD47^+^ phenotype increased with tumour progression, while no significant changes were found in the number of CSCs representing the main population of tumour cells [41]. In another study, a subpopulation of BC cells with the phenotype Oct4^hi^/CD44^hi^/med/CD24^−/+^ and CSC properties (self-renewal, cyclic rest, asymmetric division, high metastatic/invasive capacity) was also found in the bloodstream of breast cancer patients who underwent or completed treatment [157].

EMT induction tends to increase gene expression, which is associated with stemness and CSCs in some types of tumours. This has been studied using the example of normal breast tissue and BC. One of these studies showed that EMT induction in immortalised human mammary epithelial cells was sufficient to induce the expression of stem cell markers. This resulted in an increase in mammosphere formation in vitro and metastases in xenografts. Along with this, it has been shown that experimentally induced EMT increases the number of cells with stem-cell properties in mammary epithelial cells [158], and the overexpression of the transcription factors SNAI2 and SOX9 was sufficient to push the cells to acquire the stem phenotype in normal breast tissue [159]. CSCs can enter the bloodstream and become circulating tumour cells with potential metastasis to distal organs [160]. The association between CSCs and metastasis was also confirmed by the observation that disseminated cells in the bone marrow in breast cancer patients have a CSC phenotype [68]. However, this also confirms the second assumption that tumour cells in bone marrow could dedifferentiate there and become CSCs.

## 7. Importance of Tumour Cell Dedifferentiation in CSCs for Metastasis

The assumption that metastatic potential can be acquired due to the ability of non-OSCs to dedifferentiate to CSCs in the focus of possible metastasis [150] is more plausible, given the evidence of the dedifferentiation phenomenon, as one of the forms of tumour cell plasticity, characterised by the formation of CSCs from non-stem cancer cells [137]. It is important to note that dedifferentiation does not occur in normal tissue due to the formation of a large number of genetic barriers. Tumour cells can break down such barriers, and dedifferentiation is a newly acquired property of tumour cells that distinguishes them from normal cells [161], which can be used for dissemination and is quite worthy of classification as a Cancer Hallmark. It is also important to note that not all tumours within the same localization can have this property, and not all tumours are able to metastasise, even if all other links of the metastatic cascade are not disturbed [162].

On the one hand, differentiated tumour cells are capable of dedifferentiation; on the other hand, differentiated tumour cells entering the internal organs are dedifferentiated and metastasise. American scientists have presented evidence of the importance of metastasis dedifferentiation [163]. They showed that selective ablation of Lgr5^+^ tumour stem cells from colorectal cancer limits the growth of the primary tumour but does not lead to tumour regression. Instead, tumours are supported by proliferative Lgr5 cells, which try to replenish the stem cell pool. This leads to rapid reinitiation of tumour growth after treatment stops. This process is critical for the formation and growth of colorectal cancer metastases in the liver. The MYC stem gene is activated after Lgr5^+^ destroys colorectal cancer stem cells in the tumour. In a later study, it was directly established that the majority of colorectal cancer metastases were formed by Lgr5^−^ cells. These cells restored the Lgr5^+^ stem cell population, which gave rise to macrometastasis [164]. Chinese scientists discovered increased expression of Oct4 and Nanog in gefitinib-resistant NSCLC cells. They showed a multidrug resistance (MDR) phenotype and an EMT phenotype. Ectopic co-expression of Oct4/Nanog endowed NSCLC cells with CSC properties, including self-renewal, drug resistance, EMT, and high tumour-initiating activity [165].

Xu et al. (2018) believe that CSCs are not a stationary population of cells but are in dynamic homeostasis with differentiated cells. On the one hand, during asymmetric division, cancer stem cells constantly self-renew and form differentiated tumour cells. On the other hand, differentiated tumour cells are continuously dedifferentiated into stem cells for tumour growth and relapse. According to the authors, this completely cancels the therapeutic strategies aimed at destroying CSCs. Moreover, not all tumours in vivo are capable of dedifferentiation, and there is no homeostasis between non-CSCs and CSCs [166].

In our study, in the example of breast cancer, it was shown that the presence of amplified chromosomal regions with localization of stem genes in the tumour is associated with the ability to metastasise, determining the possibility of dedifferentiation of CD44^−^CD24^−^ cells of primary breast tumour cultures in CD44^+^CD24^−^ CSCs with active proliferation and mammary formation [150]. In this direction, we investigated the expression of the MYC and OCT4 proteins in subpopulations of breast tumour cells. Differentiated CD44 tumour cells with MYC and OCT4 stem protein expression are present in breast tumours. The concentration of these proteins is significantly higher in patients with metastases than in patients without metastases, while they express the EMT marker Snai2. These differentiated tumour cells with the expression of stem proteins can enter the bloodstream, and there are many more of them in patients with metastases than in patients without metastases. At the same time, CD44^–^ with the expression of stem proteins in the breast tissue undergo EMT and enter the bloodstream in the form of CTCs, which, under the influence of EMT, become CD44^+^CD24^+^Oct3^+^ progenitor cells and their high concentration is associated with metastasis [167].

So, the dynamic state and mutual transformations of CSCs to non-CSCs and non-CSCs-to-CSCs and their role in metastasis are only just beginning to be investigated, and possible therapeutic strategies that block these transitions may prove successful.

## 8. Other Forms of CSC Plasticity

Tumour cells and CSCs, in addition to dedifferentiation, are capable of a reversible transition between different cellular states, such as transdifferentiation, symmetric/asymmetric division, dormancy/proliferation, EMT/MET, and drug sensitivity/resistance [136,139,168]. Dedifferentiation occurs in the same clone, while transdifferentiation occurs between different cell clones. At the same time, aberrant activation of plasticity contributes to the appearance, maintenance, and progression of the tumour, causing aggressive behaviour of the CSCs [168]. Plasticity allows tumour cells and CSCs to switch between proliferative and calm phenotypic states, which contributes to their survival in distant organs and regulates tumour growth [169]. Highly invasive CSCs (introduced into the organ or dedifferentiated from non-CSCs) can remain dormant for several years before starting to grow [170], causing tumour relapse after therapy [171]. Internal and external factors ensure the acquisition of plasticity properties. Internal factors act through ectopic expression of transcription factors [172], stem genes [150], and EMT markers [173]. Several studies have confirmed the importance of key transcription factors, such as OCT3/4, SOX2, NANOG, and KLF4, in modulating CSC generation and regulating cell plasticity [77,143]. Overexpression of the EZH2 gene can increase the formation of mammospheres and CSC self-renewal in breast cancer [174]. Various mechanisms of epigenetic regulation have been mentioned, such as the state of bivalent chromatin, DNA methylation, and histone modifications that mediate CSC plasticity [175]. Loss of HOXC8 gene function in non-tumour epithelial cells of the mammary gland due to hypermethylation of its promoter DNA is associated with an increase in the CSC pool, increased self-renewal, and the emergence of a transformed phenotype [176]. CSCs are able to use epigenetic modifications to achieve their flexible nature [177], and this reversibility suggests an attractive potential for therapeutic targeting.

External factors are signals from the microenvironment, such as TGFb, IL6, HIF, etc. [136,178,179,180]. Doherty et al. showed that macrophages can secrete a factor such as OSM in response to chemotherapy. OCM is a cytokine of the IL-6 family that can activate the dedifferentiation of triple-negative breast cancer cells into aggressive stem cells. This activation can be mediated by the transmission of STAT3/SMAD3 signals [181].

The mechanisms of CSC chemoresistance can also be divided into two main groups: internal resistance, which is associated with genetic changes, and external resistance, which is associated with the effects of the tumour microenvironment [182]. Genetic changes in CSCs include aberrant expression of a protein that detoxifies chemotherapeutic agents. For example, ABC transporters (especially ABCG2) are usually expressed at high levels in CSCs and can cause the leakage of chemotherapy drugs from cells. However, CSCs also usually have high expression of ALDH, which metabolises chemotherapeutic agents such as cyclophosphamide and reverses the toxic effects of chemotherapy [183]. The tumour microenvironment is an important factor in CSC chemoresistance. The activation of HIF expression promotes the formation of new blood vessel and CSC phenotypes in an hypoxic environment. This phenotype contributes to CSC chemoresistance [184].

## 9. Conclusions

CSCs are an extremely heterogeneous population of cells, and no single marker for CSCs has yet been found. Different studies use different markers as CSC markers. The most studied are the CD44^+^CD24^−^, ALDH^+^ and CD133^+^ CSC phenotypes. CSCs are dynamic populations and can undergo spontaneous transitions between states and phenotypes. CSCs can give rise to various types of undifferentiated CSCs and differentiate into new tumour pseudoorganoids. In fact, tumours can function as pseudoorgans due to CSCs, also supporting the local immune system and blood supply. The environmental behaviour of CSCs includes all possible forms of positive interactions, such as commensalism, mutualism, and symbiosis, which consists of the formation of heterotypic cells with immunocytes. These forms of CSCs appear in favourable conditions for tumour development. Under unfavourable conditions, especially during the treatment of tumours, negative forms of ecological behaviour dominate: amensalism, in the form of autophagy, parasitism, in the form of induction of autophagy of neighbouring cells, and predation, in the form of cannibalism or entosis. The role of CSCs in metastasis remains controversial. There are two opposing points of view. On the one hand, metastatic cells can originate from a CSC subpopulation with the necessary genetic changes to initiate tumour growth. These cells reach secondary organs via EMT or epithelial amoeboid transition (EAT). However, no markers of metastasis have been found thus far. It is not known why CSCs with favourable conditions in the primary tumour suddenly undergo EMT or EAT migrate, penetrate into secondary organs, and form metastases. On the other hand, metastatic potential can be acquired due to the ability to dedifferentiate non-CSCs to CSCs in possible metastasis. In this case, it is not necessary to look for markers of metastasis, but markers with the ability to dedifferentiate and block metastasis based on the dedifferentiation inhibition process can be developed. This hypothesis has increasingly more evidence. In addition, there is an opinion that CSCs are not a stationary population of cells but are in dynamic homeostasis with differentiated cells. This completely reverses the therapeutic strategies aimed at destroying CSCs.

## Figures and Tables

**Table 1:** Possible CSC phenotypes of breast cancer according to the literature data.

Phenotype	Sources of Samples	Source
ABCG2^+^	Cell line HCC1937	[49]
ANTXR1^+^	Mouse mammary metastatic tumor, cell line TMD231	[50]
CD29^+^	Cell line MCF-7	[51]
CD61^+^	Mouse mammary tumor MMTV-Wnt-1	[52]
CD133^+^	Primary human breast tumor; cell lines MDA-MB-231, MCF-7, ZR-75	[53,54]
CXCR4^+^	Metastatic breast cancer; cell line MCF-7; mouse cell lines 4T1, 4T07, 168Farn, 67NR	[55]
PROCR^+^	Cell line MDA-MB-361; adipose tissue of the mammary gland of mice with MDA-MB-231	[56]
CD24^+^CD29^+^	Primary breast cancer BRCA1 mouse; mouse mammary tumor tissue MMTV-WNT1	[57]
CD24^+^CD49f^+^	BRCA1 mouse primary breast tumor	[58]
CD44^+^CD24^−/low^	Primary human breast tumor; cell lines MCF-7, BT-549, MDA-MB-231, MDA-MB-361, MDA-MB-468, T47D, ZR75, SK-BR-3, HCC1937	[44]
CD49f^hi^CD61^hi^	Transgenic mouse model HER2/neu	[59]
CD133^+^ALDH1^+^	Invasive ductal breast tumor	[60]
CD44^+^CD24^−/low^ABCG2^+^	MDA-MB-231 and MCF-7 cell lines	[61]
CD44^+^CD24^−/^^low^ALDH1^+^	Invasive ductal human breast cancer; cell lines MDA-MB-231, MDA-MB-453, MDA-MB-468, SUM149, SUM159, SK-BR-3, ZR-75, C 1954	[46,62]
CD44^+^CD24^−/^^low^EpCAM^+^	Cell lines MCF-7, MDA-MB-231, SUM149 и SUM159	[42]
CD44^+^CD24^−/^^low^SSEA-3^+^	MCF-7 and MDA-MB-231 cell lines	[63]
CD44^+^CD49f^+^CD133/2^+^	Primary ER–human breast tumor	[64]
CD44^+^CD133^+^ALDH1^+/^^hi^	Cell line MDA-MB-468	[65]
CD133^hi^CXCR4^hi^ALDH1^hi^	Invasive ductal human breast cancer	[66]
EpCAM^+^CD49f^+^	Aberrant human progenitor cells from BRCA1-mutant breast tissue	[67]
EpCAM^hi^PROCR^hi^SSEA-3^+^	MCF-7 and MDA-MB-231 cell lines	[63]
GD2^+^GD3^+^GD3S^hi^	Cell lines MDA-MB-231 and MDA-MB-468	[68]

## Data Availability

Not applicable.
